# Will Chinese external therapy with compound Tripterygium wilfordii hook F gel safely control disease activity in patients with rheumatoid arthritis: design of a double-blinded randomized controlled trial

**DOI:** 10.1186/s12906-017-1957-z

**Published:** 2017-09-05

**Authors:** Quan Jiang, Xiao-Po Tang, Xian-Chun Chen, Hong Xiao, Ping Liu, Juan Jiao

**Affiliations:** 1 Rheumatism Department, Guang’anmen Hospital, China Academy of Chinese Medical Sciences, No. 5 Beixiange Street, Xicheng District, Beijing, 100053 China; 2 Pharmaceutical Department, Guang’anmen Hospital, China Academy of Chinese Medical Sciences, No. 12 Fuhai Street, Daxing District, Beijing, 102628 China; 3 Clinical evaluation center, Guang’anmen Hospital, China Academy of Chinese Medical Sciences, No. 5 Beixiange Street, Xicheng District, Beijing, 100053 China

**Keywords:** Chinese external therapy, Topical application, Tripterygium, Rheumatoid arthritis

## Abstract

**Background:**

Chinese external therapy (CET) is a topical application with mainly Chinese herb medicine therapy with thousands of years of historical implications and is a clinical routine that is commonly used for relieving joint-related symptoms in patients with arthritis in Chinese hospitals. However, there is a paucity of modern medical evidence to support its effectiveness and safety. Thus, we propose to implement a randomized, double-blinded, placebo-controlled clinical trial in patients with rheumatoid arthritis (RA) using, as the experimental intervention, topical application of a hospital-compounded gel preparation of Tripterygium wilfordii Hook F (TwHF).

**Methods:**

This study will be an 8-week double-blinded, randomized, placebo-controlled clinical trial conducted at Guang’anmen Hospital in Beijing, China, and 168 patients with moderately active RA will be randomly assigned with a 1:1 ratio to apply a topical gel preparation containing TwHF or placebo. The primary outcome variable will be the proportion of subjects, by study group, to achieve a 20% improvement in the American College of Rheumatology criteria (ACR20) by week 8. Secondary outcome measures to be assessed at weeks 4 or 8 will include: measurement of ACR20 response rate at week 4, ACR50 response rate, the changes in DAS28 score, and joint synovitis classification assessment monitored by musculoskeletal ultrasound. Safety evaluations conducted at weeks 4, 8 and 12 will be based on spontaneous complaints by the study subjects, but special emphasis will be focused on cutaneous allergy and alterations of menstruation in premenopausal female participants. Statistical analyses will be performed using the intention to treat analysis data set.

**Discussion:**

This proposed clinical trail is designed to evaluate the efficacy and safety of CET based on a single topically-applied agent in a relatively large patient population with RA. This study protocol gives a detailed description of the usage and dosage of the topical compound TwHF gel and the methodology of this study. In addition, it is hoped that the outcomes of this study will be viewed as supporting the generalizability of CET in the setting of inflammatory rheumatic diseases. The results of this study are expected to have important public health implications for Asian RA patients that currently utilize CET as a complimentary treatment.

**Trial registration:**

Clinical trial gov Identifier: NCT02818361. Registrated on Jun. 15, 2016.

## Background

Rheumatoid arthritis (RA) is a chronic, systemic disorder characterized by persistent synovitis involving diarthrodial joints. Uncontrolled active RA can result in joint damage, disability, decreased quality of life, cardiovascular disease, and other comorbidities. Although Non-Steroid Anti-Inflammatory Drugs (NSAIDs) and steroids have been recommended by many physicians to relieve joint pain, and other symptoms related to the chronic synovitis and systemic inflammation, those drugs carry an increased potential for side effects [1] and are poorly tolerated by many patients.

For centuries in China, Chinese external therapy (CET) has been an accepted clinical approach for relieving joint-related symptoms and has been well-received by Chinese RA patients. In a narrow sense, CET is a pharmacological treatment for body surface or skin (mucosa) diseases, such as fumigation and plaster, etc.. CET has a long history that has its origins in the earliest experience of Chinese Medicine during the Chinese Qin and Han dynasties (BCE 221 to CE 220). It was observed that RA symptoms typically present as the swelling of multiple limb joints, pain, and stiffness, the main presenting clinical manifestations of active RA exist near the skin surface. CET is believed to have a rapid and favorable effect because it can be applied directly above the lesions. However, there are very few modern evidence-based data to support its effectiveness and safety in active rheumatic diseases.

Tripterygium wilfordii Hook F (TwHF), a traditional Chinese herb, has been used to relieve joint pain for a thousand years in China and its extracts have recently proven to be effective in patents with RA [2, 3]. However, the oral application of TwHF is limited for clinical use because of its high frequency of reproductive system damage [4]. In order to reduce toxicity without losing efficacy, the current study utilizes a topical rather than an oral compound THwF, hoping to thereby explore the advantages of CET. We previously conducted a 4-week double-blinded, randomized, placebo-controlled pilot clinical trial on topical compound TwHF with 70 RA patients whose joint disease was severely-active. The findings of the pilot study showed that joints pain and swelling were rapidly (within one week) reduced with the application of topical compound TwHF twice a day. Furthermore, a favorable control of the overall RA disease condition was found, as the rate of achievement rose to 20% and 50% improvement in the (ACR20 and ACR50) were 34% and 17% at week 4, using intention to treat analysis [currently awaiting publication]. The ACR20 and ACR50 response rates were found to be even higher in another study which reported responses of 58% and 20% with the TwHF group at week 6 [5]. Additionally, a previous double-blind, randomized multicenter trial found that based upon methotrexate or leflunomide, 75 out of 87 patients (90.8%) in the topical compound TwHF group and 58 out of 87 patients (69.0%) in the topical placebo-treated group reported relief from their joint pain [6].

Encouraged by the above results, we have developed the topical compound TwHF to be used as a hospital gel preparation. Thus, we plan to undertake an 8-week double-blinded randomized placebo-controlled clinical trial to further explore the safety and effectiveness of topical TwHF gel in patients with active RA.

It is important to examine whether topical treatment, with TwHF gel, as a supplement to usual care, will result in better outcomes for patients with active RA. We expect a favorable outcome for the topical compound TwHF gel that supports its efficiency. This article provides a full description of the study’s design and methodology in accordance with the SPIRIT guidelines for reporting protocols for intervention trials and the CONSORT guidelines [7, 8] for reporting outcomes.

## Methods

### Study design

This clinical trail will be accomplished at Guang’anmen hospital, China academy of Chinese Medical Science in Beijing, China. The current study follows the guidelines of the Declaration of Helsinki for humans and has been approved by the ethic s committee of Guang’anmen Hospital (approval number: 2016–062-KY). Enrolled will be 168 patients with documented moderately active RA who will be randomized (1:1 ratio) to one of two treatment groups: the TwHF gel group and the placebo gel group. Written signed consent will be obtained from each participant before any study-related procedures are initiated.

### Participants

#### Inclusion criteria

Eligible patients, men and women, must age from 18 to 65 years old, and must be clinically diagnosed with RA prior to screening for study entry in accordance with the 1987 revised criteria of the American College of Rheumatology [9]. Their RA disease stage must be in the moderate activity range, as defined by the criteria of the 28-joint count Disease Activity Score (DAS-28 score), ranging between 3.2 to 5.1 [10]. If patients are receiving a disease-modifying, anti-rheumatic drugs (DMARDs), those medications should be continued unchanged throughout this study, but their dosages must have been stable for at least 12 weeks prior to study entry.

#### Exclusion criteria

Exclusion Criteria will include: ongoing rheumatic or inflammatory joint diseases other than RA; serious medical diseases such as: hyperlipidemia, hyperglycemia, diabetes mellitus, cardiovascular diseases, gastrointestinal disease, liver disease or renal failure; patients with RA who have a prominent component of comorbid fibromyalgia syndrome, prior treatment with TwHF agents, glucocorticoids, or biological agents; skin allergies or broken skin in the areas of planned treatment with topical TwHF gel. Female patients who are pregnant, breast-feeding or who plan to become pregnant will be excluded.

### Randomization

Randomization will be controlled by an independent third party, the hospital Clinical Evaluation Center, using the SAS system (Version 8.2 for Windows) to assign the eligible participants at a 1:1 ratio to receive either topical compound TwHF gel or placebo.

### Blinding

This study is a double-blind and placebo-controlled design. The blinding will also be accomplished and monitored by the hospital Clinical Evaluation Center. The blinding codes record topical compound TwHF gel and the placebo as A and B, sequentially and the details of the allocation sequence will be put in sealed light-tight envelopes and maintained in the Clinical Evaluation Center. The contents of these envelopes will be unknown to the principal investigator, the study staff, and the participants. Since the packing and labeling of the treatment gels are the same, the topical compound TwHF gel and placebo gel will be masked by numeric codes from 001 to 168, which correspond with each participant’s sequential enrollment number. Thus, the study researchers will enroll and evaluate study subjects but will have no influence on randomization. So the case group division is adapted to a randomized, double-blind study design.

### Intervention

The ingredients for the compounding of the topical TwHF gel include: Tripterygium wilfordii Hook F, Mangxiao (Mirabilite), Chuanxiong (Rhizoma Ligustici), Ruling (Olibanum), and Moyao (M yrrh) (proportions: 4:4:2:2:1). The topical placebo gel is made of a viscous agent and sucrose which make it looks like the active preparation. Both of the gels will be prepared by the Pharmaceutical Department of Guang’anmen Hospital, and the herbs will be purchased from Beijing Fengtaijinyuan Pharmaceutical Co., Ltd. Each gel is 20 gram (g) per tube. Both the topical compound TwHF gel and the placebo gel will be applied for 1st to 5th metacarpophalangeal joints, 1st to 5th proximal interphalangeal joints, wrists, knees and ankles 10 g for 1 h, once a day from week 0 through week 4 and 10 g from week 5 through week 8.

### Outcome measurements

Outcomes measurements will be assessed at baseline, at week 4, at week 8 and at the 12th week follow-up. The DAS28 joints will be assessed for swelling and tenderness by a trained staff member. All the outcome assessments will be accomplished by the same staff researcher.

The primary outcome measure will be ACR20 criteria [11] by week 8. To achieve ACR20, a patient must have a 20% or greater reduction in the number of both tender and swollen joints and a 20% or more improvement in 3 or more of the following: the physician’s or patient’s assessment of global health status, the patient’s assessment of pain on a visual analogue scale, the patient’s assessment of function using a modified version of the Health Assessment Questionnaire (HAQ), and the erythrocyte sedimentation rate (ESR) or serum C-reactive protein (CRP) level.

Secondary outcome measures will include: ACR20 assessed at week 4, ACR50 criteria assessed at weeks 4 and 8, changes at weeks 4 and 8 in DAS28, and joint synovitis classification. Since the DAS28 is a combined index to measure the disease activity in patients with RA, it can be used in combination with the European League Against Rheumatism response criteria [10]. In the present trial, the DAS28 score will be calculated, based on either the C-reactive protein (DAS28-CRP) or the erythrocyte sedimentation rate (DAS28-ESR) [10]. Changes in joint synovitis classification will be determined by serial musculoskeletal ultrasound (MUSU) focused on the total sum of synovitis classification based on the joint used gels. The MUSU synovitis classification will derive from each MUSU measurement as follows: 0 level, no doppler synovial tissue signal; level 1, three independent points or 2 successive or 1 to 2 independent dot doppler synovial tissue signals; level 2, doppler signal indicating <50% of the area representing synovium; level 3, doppler signal indicating synovium in >50% of the measured area [12].

The safety evaluation will be accomplished by a face-to-face interview at the 4th and 8th weeks and by a a telephone-interview with the remote 12th week follow-up. Adverse experiences of interest will include: manifestations of cutaneous allergy (erythema, edema, itching) and gastrointestinal stimulation due to mucous membrane damage. In addition, because of the high frequency of reproductive system damage, the safety evaluation will focus on changes in menstruation patterns among premenopausal female participants. The relevant female participants will be asked a general question about whether they have observed any change in their menstruation pattern. Irrespective of how they answer that question, a series of follow-up questions will attempt to quantify any change in the menstrual cycle (extension, shortening or no change), or in the quantity of menstrual flow (increased, reduced, or unchanged). Blood and urine routine laboratory examinations, including glutamic-pyruvic transaminase, glutamic oxalacetic transaminase, creatinine and urea nitrogen will be accomplished at baseline and on the 8th week. Quantitative changes will be addressed on the record. Professional judgments regarding the severity of any given adverse effect and any recognized relationship between the adverse effect and the study interventions will be documented if any of the above safety measures are observed to fall beyond normal clinical or laboratory ranges.

### Sample size

This study is designed as a superiority trial. A previous findings showed the ACR20 response rates were 34.3% in a topical TwHF group and 12.5% in placebo group in patients with active RA [awaiting publication]. Based on the following calculation: *n* = 2(u_α_ + u_β)_^2^2P(1-P)/(P_1_-P_0_)^2^ [13], it is predicted that 76 participants in each group will be needed to detect a significant (p < =0.05) with 80% power. Based upon a predicted 10% rate of drop out, we will recruit a total of 168 participants, 84 in each group.

### Quality control

The identification, registration, and subsequent flow of participants in the trial will be governed by the trial standard operational protocol. The completion of the Case Report Form and compliance with the standard operation procedures will be audited. At regular intervals, a clinical research associate, will monitor the clinical trial procedures, such as compliance with the study protocol and regulatory policy. In particular, the reasons for withdrawal will be fully documented in the Case Report Form.

### Data analysis

The statistical analyses will be performed using the intention to treat analysis (ITT). The safety dataset will also include all patients who received ≥1 week of topical applications. Mean ± Standard Deviation, number and percentage will be used for continuous variables and categorical variables, as appropriate. Patient movement through the study will be documented on a Consort diagram. Patient demographic characteristics will be documented in a by-group comparison table and statistically compared between topical TwHF and placebo groups using a 2-sample t test for continuous variables or Pearson 2 test for categorical variables.

All analyses will be performed using SAS system (Version 5.2.127 8.2 for Windows). *P* values less than .05 will be considered statistically significant. The primary outcome variable, the ACR20 response rate at week 8, and the secondary outcome measure, ACR20 at week 4 and ACR 50 response rate at weeks 4 and 8, will be compared between the 2 groups using the Pearson 2 test. Two-sample t tests will be performed on DAS28-CRP and DAS28-ESR at baseline and posttreatment, as well as their changes after treatment. Paired t tests will be used to compare the changes of DAS28-CRP and DAS28-ESR from baseline to posttreatment in each group. The joint synovitis classification will be compared between the 2 groups at baseline and posttreatment using Kruskal-Wallis rank sum test. Any by-group changes in the joint synovitis classification will be compared using the paired Kruskal-Wallis rank sum test.

### Ethical considerations

This study has been approved by the ethics committee of Guang’anmen Hospital (approval number: 2016–062-KY). Written consent to take part in the study will be obtained from all study participants before any of the study-related procedures are initiated. Each study subject will be provided a witnessed copy of the consent document he or she signed, which also bears the signature a study investigator. They will also receive an information sheet explaining the study.

## Discussion

To date, only a small number of studies have documented the effectiveness of CET using any pharmacological agent. This clinical trial is designed as a double-blinded, randomized, controlled study of TwHF-based CET in a moderate active RA population with 8 weeks of observation. Considering the main clinical features of active RA to be joint swelling, tenderness, and pain, these are the clinical manifestations monitored using validated instruments. It is anticipated that topical compound TwHF gel will also be effective in controlling the clinical manifestations of RA and safe for use by RA patients.

In animal studies, TwHF microemulsion has been observed to imeliorate the severity of a murine adjuvant-induced arthritis model and to reduce the male reproductive toxicity and hepatotoxicity [14]. It is hoped that the topical use of TwHF as a clinical application of CET in RA patients will be found to be both safe and efficacious. Although TwHF and its extracts have shown good responses in patients with RA it is currently limited to use in elderly patients because of demonstrated reproductive toxicity. Safety observations from previous studies have shown that compound TwHF applied topically can cause cutaneous reactions which are reversible with discontinuation of the topical TwHF compound. In addition, increases in serum aminotransferase and alterations in menstruation among female patients have been reported [5, 6].

There are several aspects of the current study design that are different from previous CET clinical studies using this agent. Firstly, the design of dosage adjustment in this study will demonstrate whether the period of dosage reduction will bring additional benefits to the patients. In clinical practice, patients often continue to apply the effective medicine for an additional period in order to consolidate the efficacy, though there is no evidence to support this practice. Secondly, the follow-up assessment has rarely been included in CET research. Since oral preparations of THwF have often exhibited side effects, such as gastrointestinal and menstrual disorders, special emphasis has been placed upon an 4-week follow-up (to monitor at least one menstrual cycle).

In conclusion, the successful completion of this study will contribute to the evidence base of whether TwHF compound gel is a promising complimentary treatment as a simple, inexpensive, effective and safe treatment for active RA patients. The results of this study are expected to have important public health implications which may alter the approach to management of RA in China.

## Abbreviations

ACR20: 20% improvement in the American College of Rheumatology criteria
ACR50: 50% improvement in the American College of Rheumatology criteria
CET: Chinese external therapy
CRP: C-reactive protein
DAS28: 28-joint count Disease Activity Score
DAS28-CRP: DAS28 score based on C-reactive protein
DAS28-ESR: DAS28 score based on erythrocyte sedimentation rate
DMARDs: Disease-modifying, anti-rheumatic drugs
ESR: Erythrocyte sedimentation rate
G: Gram
HAQ: Health Assessment Questionnaire
ITT: Intention to treat analysis
MU SU: Musculoskeletal ultrasound
NSAIDs: Non-Steroid Anti-Inflammatory Drugs
RA: Rheumatoid arthritis
TwHF: Tripterygium wilfordii Hook F
gram: g
